# Supplementary material for: Maternal effects, reciprocal differences and combining ability study for yield and its component traits in maize (Zea mays L.) through modified diallel analysis
Source: PeerJ. 2024 Jun 25;12:e17600. doi: 10.7717/peerj.17600 (PMC11212646; doi:10.7717/peerj.17600)
Supplement: Supplemental Information 1 [file peerj-12-17600-s001.docx]

**S1 Table. Mean performances of straight crosses**

| **Crosses** | **DTT** | **DTS** | **NKRC** | **NKR** | **CL** | **CG** | **HGW** | **GY** |
| --- | --- | --- | --- | --- | --- | --- | --- | --- |
| **1x2** | 62.00 | 62.33 | 13.80 | 34.50 | 16.30 | 4.20 | 26.50 | 56.72 |
| **1x3** | 61.67 | 63.67 | 13.40 | 32.60 | 15.52 | 4.20 | 30.00 | 39.81 |
| **1x4** | 62.00 | 62.67 | 13.80 | 28.60 | 15.65 | 4.30 | 30.50 | 58.37 |
| **1x5** | 61.67 | 63.67 | 13.20 | 29.60 | 13.60 | 4.17 | 27.00 | 38.59 |
| **1x6** | 59.33 | 59.67 | 14.80 | 35.10 | 17.05 | 4.29 | 26.50 | 54.43 |
| **1x7** | 61.33 | 62.00 | 15.20 | 34.40 | 17.15 | 4.33 | 27.50 | 70.95 |
| **1x8** | 60.33 | 63.00 | 14.00 | 35.20 | 17.65 | 4.35 | 32.00 | 57.70 |
| **2x3** | 62.33 | 61.00 | 14.60 | 36.70 | 16.15 | 4.53 | 30.50 | 44.90 |
| **2x4** | 63.67 | 63.00 | 14.80 | 34.90 | 16.87 | 4.53 | 32.00 | 53.77 |
| **2x5** | 63.00 | 63.33 | 14.80 | 35.90 | 16.32 | 4.70 | 31.50 | 44.41 |
| **2x6** | 63.33 | 63.00 | 14.60 | 36.80 | 17.10 | 4.38 | 29.50 | 47.23 |
| **2x7** | 63.33 | 63.33 | 15.20 | 38.20 | 18.10 | 4.50 | 30.00 | 31.21 |
| **2x8** | 64.00 | 63.67 | 13.60 | 33.10 | 16.00 | 4.32 | 29.50 | 31.89 |
| **3x4** | 60.33 | 60.67 | 13.20 | 31.40 | 14.65 | 4.27 | 29.50 | 52.42 |
| **3x5** | 60.67 | 62.33 | 13.27 | 26.73 | 13.13 | 4.33 | 30.50 | 48.51 |
| **3x6** | 59.33 | 60.33 | 14.20 | 34.20 | 15.65 | 4.23 | 26.00 | 55.29 |
| **3x7** | 60.67 | 61.33 | 14.40 | 36.10 | 15.70 | 4.25 | 28.00 | 52.74 |
| **3x8** | 60.00 | 63.00 | 14.80 | 31.50 | 15.25 | 4.28 | 29.00 | 42.63 |
| **4x5** | 65.00 | 66.00 | 13.60 | 29.60 | 15.10 | 4.59 | 32.00 | 35.51 |
| **4x6** | 64.33 | 64.33 | 14.20 | 35.50 | 18.26 | 4.43 | 31.50 | 63.84 |
| **4x7** | 63.67 | 62.67 | 16.00 | 37.00 | 18.15 | 4.60 | 31.00 | 86.41 |
| **4x8** | 63.33 | 63.67 | 14.47 | 34.93 | 18.15 | 4.60 | 36.00 | 42.74 |
| **5x6** | 62.33 | 63.00 | 15.20 | 33.00 | 17.21 | 4.82 | 31.00 | 86.12 |
| **5x7** | 61.67 | 62.67 | 14.20 | 36.90 | 16.85 | 4.60 | 30.00 | 71.23 |
| **5x8** | 65.67 | 65.00 | 14.00 | 28.20 | 14.45 | 3.88 | 28.00 | 33.09 |
| **6x7** | 66.33 | 66.67 | 14.20 | 30.60 | 13.30 | 4.22 | 25.50 | 36.83 |
| **6x8** | 64.00 | 65.33 | 14.20 | 32.40 | 16.60 | 4.24 | 30.00 | 46.10 |
| **7x8** | 64.67 | 65.33 | 15.80 | 38.00 | 18.79 | 4.65 | 30.50 | 49.35 |
