# Supplementary material for: Maternal effects, reciprocal differences and combining ability study for yield and its component traits in maize (Zea mays L.) through modified diallel analysis
Source: PeerJ. 2024 Jun 25;12:e17600. doi: 10.7717/peerj.17600 (PMC11212646; doi:10.7717/peerj.17600)
Supplement: Supplemental Information 2 [file peerj-12-17600-s002.docx]

**S2 Table. Mean performances of reciprocal crosses**

| **Crosses** | **DTT** | **DTS** | **NKRC** | **NKR** | **CL** | **CG** | **HGW** | **GY** |
| --- | --- | --- | --- | --- | --- | --- | --- | --- |
| **2x1** | 62.33 | 62.67 | 13.80 | 37.70 | 20.08 | 4.69 | 35.50 | 83.04 |
| **3x1** | 59.67 | 58.67 | 13.00 | 37.20 | 18.10 | 4.40 | 34.50 | 44.45 |
| **4x1** | 63.00 | 62.67 | 13.60 | 34.10 | 17.09 | 4.47 | 32.50 | 43.80 |
| **5x1** | 60.33 | 62.00 | 13.80 | 34.40 | 16.01 | 4.65 | 30.50 | 51.53 |
| **6x1** | 66.00 | 66.00 | 12.80 | 30.90 | 16.52 | 3.98 | 27.50 | 36.12 |
| **7x1** | 64.67 | 65.00 | 14.80 | 29.90 | 14.55 | 4.63 | 28.50 | 43.80 |
| **8x1** | 59.67 | 61.67 | 13.80 | 37.90 | 18.40 | 4.59 | 36.50 | 86.81 |
| **3x2** | 59.33 | 61.00 | 14.00 | 31.70 | 14.66 | 4.24 | 28.00 | 65.41 |
| **4x2** | 63.00 | 62.00 | 14.40 | 32.90 | 16.00 | 4.33 | 28.50 | 42.10 |
| **5x2** | 62.67 | 62.67 | 15.70 | 38.60 | 17.85 | 4.94 | 35.00 | 88.22 |
| **6x2** | 64.33 | 63.67 | 13.80 | 35.90 | 15.90 | 4.34 | 30.00 | 49.31 |
| **7x2** | 63.33 | 63.33 | 15.60 | 34.00 | 16.24 | 4.44 | 29.00 | 46.52 |
| **8x2** | 63.67 | 64.33 | 13.80 | 28.20 | 13.55 | 3.89 | 27.50 | 34.26 |
| **4x3** | 61.67 | 62.00 | 14.00 | 32.60 | 14.66 | 4.46 | 29.50 | 52.42 |
| **5x3** | 62.00 | 63.33 | 15.40 | 40.00 | 18.40 | 5.01 | 35.00 | 80.81 |
| **6x3** | 64.00 | 65.00 | 14.80 | 31.00 | 16.28 | 4.17 | 26.50 | 47.94 |
| **7x3** | 63.00 | 64.00 | 14.40 | 35.50 | 17.20 | 4.33 | 28.00 | 73.78 |
| **8x3** | 64.00 | 65.67 | 14.00 | 37.20 | 17.35 | 4.46 | 34.00 | 60.91 |
| **5x4** | 63.67 | 63.33 | 14.40 | 33.90 | 18.00 | 5.13 | 39.00 | 86.56 |
| **6x4** | 63.67 | 63.33 | 13.60 | 30.80 | 16.45 | 4.24 | 30.50 | 50.09 |
| **7x4** | 63.33 | 62.33 | 16.00 | 38.00 | 19.68 | 4.74 | 32.50 | 88.90 |
| **8x4** | 63.67 | 63.67 | 13.60 | 33.60 | 17.50 | 4.43 | 35.00 | 53.43 |
| **6x5** | 63.33 | 63.33 | 15.40 | 33.00 | 16.33 | 4.59 | 31.50 | 31.19 |
| **7x5** | 64.00 | 64.00 | 16.00 | 35.40 | 17.75 | 4.83 | 31.50 | 59.88 |
| **8x5** | 65.33 | 65.00 | 13.60 | 29.40 | 15.15 | 3.79 | 26.00 | 30.75 |
| **7x6** | 64.67 | 64.33 | 14.60 | 37.10 | 17.55 | 4.41 | 27.00 | 56.25 |
| **8x6** | 64.33 | 64.67 | 14.00 | 35.00 | 17.25 | 4.38 | 32.00 | 55.86 |
| **8x7** | 65.00 | 65.33 | 15.80 | 36.30 | 18.70 | 4.51 | 31.00 | 44.15 |
