# Supplementary material for: Maternal effects, reciprocal differences and combining ability study for yield and its component traits in maize (Zea mays L.) through modified diallel analysis
Source: PeerJ. 2024 Jun 25;12:e17600. doi: 10.7717/peerj.17600 (PMC11212646; doi:10.7717/peerj.17600)
Supplement: Supplemental Information 3 [file peerj-12-17600-s003.docx]

**S3 Table. Mid-parent heterosis of straight crosses**

| **Crosses** | **DTT** | **DTS** | **NKRC** | **NKR** | **CL** | **CG** | **HGW** | **GY** |
| --- | --- | --- | --- | --- | --- | --- | --- | --- |
| **1x2** | -4.62 | -5.87 | 11.29 | 76.92 | 44.89 | 18.14 | 45.21 | 135.68 |
| **1x3** | 1.93 | -1.80 | 5.51 | 36.12 | 22.45 | 12.90 | 76.47 | 38.00 |
| **1x4** | -5.82 | -8.51 | 11.29 | 42.64 | 44.57 | 23.39 | 84.85 | 343.17 |
| **1x5** | -7.04 | -7.72 | 4.35 | 14.84 | 1.12 | 2.71 | 22.73 | 148.14 |
| **1x6** | -11.00 | -13.31 | 16.54 | 46.56 | 29.66 | 11.57 | 32.50 | 93.98 |
| **1x7** | -8.46 | -10.37 | 13.43 | 39.43 | 31.92 | 13.20 | 50.68 | 176.32 |
| **1x8** | -9.95 | -9.79 | 18.64 | 89.25 | 56.54 | 35.73 | 93.94 | 300.32 |
| **2x3** | 0.27 | -5.18 | 6.57 | 50.10 | 32.65 | 21.94 | 37.08 | 17.36 |
| **2x4** | -5.68 | -7.35 | 10.45 | 69.83 | 63.39 | 30.17 | 47.13 | 138.08 |
| **2x5** | -7.35 | -7.55 | 8.42 | 36.63 | 26.02 | 15.91 | 15.60 | 77.90 |
| **2x6** | -7.32 | -7.81 | 6.57 | 50.51 | 35.18 | 14.06 | 16.83 | 26.03 |
| **2x7** | -7.77 | -7.78 | 5.56 | 52.19 | 44.80 | 17.80 | 27.66 | -11.07 |
| **2x8** | -6.80 | -8.17 | 6.25 | 73.30 | 48.49 | 34.94 | 35.63 | 33.83 |
| **3x4** | -4.23 | -7.61 | -3.65 | 25.60 | 24.68 | 17.15 | 43.90 | 91.56 |
| **3x5** | -4.46 | -5.80 | -4.90 | -13.00 | -8.66 | 2.61 | 17.31 | 63.12 |
| **3x6** | -7.05 | -8.59 | 1.43 | 18.34 | 11.19 | 5.49 | 8.33 | 30.85 |
| **3x7** | -5.45 | -7.55 | -2.04 | 22.17 | 12.75 | 6.65 | 25.84 | 32.28 |
| **3x8** | -6.49 | -5.97 | 12.98 | 33.76 | 25.00 | 27.19 | 41.46 | 49.03 |
| **4x5** | -5.57 | -5.48 | -0.37 | 10.14 | 20.56 | 15.18 | 25.49 | 152.43 |
| **4x6** | -6.99 | -7.66 | 3.65 | 76.62 | 49.37 | 17.51 | 34.04 | 140.21 |
| **4x7** | -8.39 | -10.47 | 11.11 | 79.61 | 50.31 | 22.67 | 42.53 | 257.16 |
| **4x8** | -8.87 | -9.90 | 13.02 | 39.44 | 75.33 | 46.86 | 80.00 | 230.59 |
| **5x6** | -10.53 | -10.21 | 8.96 | 28.03 | 15.89 | 10.93 | 6.90 | 197.42 |
| **5x7** | -11.90 | -11.11 | -3.07 | 40.44 | 14.63 | 6.36 | 10.09 | 168.06 |
| **5x8** | -6.19 | -8.66 | 7.28 | -8.22 | 11.37 | 4.72 | 9.80 | 116.22 |
| **6x7** | -5.69 | -5.21 | -3.40 | 25.15 | -7.64 | 2.68 | 0.99 | -5.76 |
| **6x8** | -9.00 | -7.99 | 8.40 | 12.11 | 30.97 | 21.49 | 27.66 | 65.73 |
| **7x8** | -8.49 | -8.42 | 14.49 | 57.02 | 50.02 | 34.01 | 40.23 | 94.03 |
