# Supplementary material for: Maternal effects, reciprocal differences and combining ability study for yield and its component traits in maize (Zea mays L.) through modified diallel analysis
Source: PeerJ. 2024 Jun 25;12:e17600. doi: 10.7717/peerj.17600 (PMC11212646; doi:10.7717/peerj.17600)
Supplement: Supplemental Information 4 [file peerj-12-17600-s004.docx]

**S4 Table. Mid-parent heterosis of reciprocal crosses**

| **Crosses** | **DTT** | **DTS** | **NKRC** | **NKR** | **CL** | **CG** | **HGW** | **GY** |
| --- | --- | --- | --- | --- | --- | --- | --- | --- |
| **2x1** | -4.10 | -6.70 | 11.29 | 93.33 | 78.49 | 31.93 | 94.52 | 245.05 |
| **3x1** | -1.38 | -9.51 | 2.36 | 55.32 | 42.8 | 18.28 | 102.94 | 54.11 |
| **4x1** | -4.3 | -8.51 | 9.68 | 69.65 | 57.88 | 28.26 | 96.97 | 436 |
| **5x1** | -9.05 | -10.14 | 9.09 | 33.46 | 19.03 | 14.53 | 38.64 | 231.42 |
| **6x1** | -1.00 | -4.12 | 0.79 | 29.02 | 25.63 | 3.51 | 37.5 | 28.74 |
| **7x1** | -3.48 | -6.03 | 10.45 | 21.54 | 11.92 | 21.05 | 56.16 | 70.59 |
| **8x1** | -10.95 | -11.69 | 16.95 | 103.76 | 63.19 | 43.21 | 121.21 | 502.22 |
| **3x2** | -4.56 | -5.18 | 2.19 | 29.65 | 20.41 | 14.13 | 25.84 | 70.97 |
| **4x2** | -6.67 | -8.82 | 7.46 | 59.71 | 54.96 | 24.43 | 31.03 | 86.41 |
| **5x2** | -7.84 | -8.51 | 15.02 | 46.91 | 37.84 | 21.82 | 28.44 | 253.41 |
| **6x2** | -5.85 | -6.83 | 0.73 | 46.83 | 25.69 | 13.02 | 18.81 | 31.59 |
| **7x2** | -7.77 | -7.78 | 8.33 | 35.46 | 29.92 | 16.23 | 23.4 | 32.56 |
| **8x2** | -7.28 | -7.22 | 7.81 | 47.64 | 25.75 | 21.56 | 26.44 | 43.79 |
| **4x3** | -2.12 | -5.58 | 2.19 | 30.14 | 24.77 | 22.36 | 43.90 | 91.58 |
| **5x3** | -2.36 | -4.28 | 10.39 | 30.19 | 28.00 | 18.72 | 34.62 | 171.74 |
| **6x3** | 0.26 | -1.52 | 5.71 | 7.27 | 15.67 | 4.12 | 10.42 | 13.46 |
| **7x3** | -1.82 | -3.52 | -2.04 | 20.14 | 23.52 | 8.66 | 25.84 | 85.06 |
| **8x3** | -0.26 | -1.99 | 6.87 | 57.96 | 42.21 | 32.54 | 65.85 | 112.95 |
| **5x4** | -7.51 | -9.31 | 5.49 | 26.14 | 43.71 | 28.73 | 52.94 | 515.37 |
| **6x4** | -7.95 | -9.09 | -0.73 | 22.95 | 34.56 | 12.47 | 29.79 | 88.45 |
| **7x4** | -8.87 | -10.96 | 11.11 | 47.86 | 62.98 | 26.13 | 49.43 | 267.44 |
| **8x4** | -8.39 | -9.90 | 6.25 | 70.56 | 69.08 | 41.53 | 75.00 | 313.24 |
| **6x5** | -9.09 | -9.74 | 10.39 | 7.40 | 9.97 | 5.64 | 8.62 | 7.71 |
| **7x5** | -8.57 | -9.22 | 9.22 | 12.83 | 20.75 | 11.68 | 15.60 | 125.35 |
| **8x5** | -6.67 | -8.66 | 4.21 | 15.86 | 16.76 | 2.29 | 1.96 | 100.9 |
| **7x6** | -8.06 | -8.54 | -0.68 | 25.55 | 21.88 | 7.30 | 6.93 | 43.93 |
| **8x6** | -8.53 | -8.92 | 6.87 | 48.62 | 36.09 | 25.5 | 36.17 | 100.81 |
| **8x7** | -8.02 | -8.42 | 14.49 | 50.00 | 49.30 | 29.97 | 42.53 | 73.57 |
