# Supplementary material for: Maternal effects, reciprocal differences and combining ability study for yield and its component traits in maize (Zea mays L.) through modified diallel analysis
Source: PeerJ. 2024 Jun 25;12:e17600. doi: 10.7717/peerj.17600 (PMC11212646; doi:10.7717/peerj.17600)
Supplement: Supplemental Information 5 [file peerj-12-17600-s005.docx]

**S5 Table. Better parent heterosis of straight crosses**

| **Crosses** | **DTT** | **DTS** | **NKRC** | **NKR** | **CL** | **CG** | **HGW** | **GY** |
| --- | --- | --- | --- | --- | --- | --- | --- | --- |
| **1x2** | -3.13 | -5.16 | 2.99 | 72.50 | 38.72 | 17.98 | 12.77 | 69.42 |
| **1x3** | 8.19 | 2.69 | -4.29 | 12.80 | 14.12 | 8.25 | 42.86 | -7.50 |
| **1x4** | -3.13 | -7.39 | 2.99 | 35.55 | 33.19 | 20.79 | 52.50 | 399.38 |
| **1x5** | -3.65 | -5.91 | -5.04 | -9.06 | -10.23 | -8.55 | -12.90 | 134.65 |
| **1x6** | -7.29 | -11.82 | 5.71 | 21.45 | 17.18 | 3.87 | -1.85 | 31.27 |
| **1x7** | -4.17 | -8.38 | -1.30 | 13.58 | 20.35 | 5.87 | 17.02 | 93.33 |
| **1x8** | -5.73 | -6.90 | 14.75 | 85.26 | 50.21 | 22.19 | 60.00 | 307.16 |
| **2x3** | 9.36 | -1.61 | 4.29 | 26.99 | 18.75 | 16.75 | 29.79 | 4.33 |
| **2x4** | -3.54 | -5.50 | 10.45 | 65.40 | 56.93 | 27.61 | 36.17 | 60.60 |
| **2x5** | -4.55 | -5.01 | 6.47 | 10.29 | 7.72 | 3.07 | 1.61 | 32.63 |
| **2x6** | -4.04 | -5.50 | 4.29 | 27.34 | 17.53 | 6.05 | 9.26 | 13.89 |
| **2x7** | -4.04 | -5.01 | -1.30 | 26.49 | 27.02 | 10.02 | 27.66 | -14.97 |
| **2x8** | -3.03 | -4.50 | 1.49 | 65.50 | 48.15 | 21.63 | 25.53 | -4.76 |
| **3x4** | 5.85 | -2.15 | -5.71 | 8.65 | 7.72 | 10.05 | 40.48 | 21.80 |
| **3x5** | 6.43 | 0.53 | -5.24 | -17.88 | -13.33 | -5.04 | -1.61 | 12.72 |
| **3x6** | 4.09 | -2.69 | 1.43 | 18.34 | 7.56 | 2.30 | -3.70 | 28.46 |
| **3x7** | 6.43 | -1.08 | -6.49 | 19.54 | 10.18 | 3.91 | 19.15 | 22.54 |
| **3x8** | 5.26 | 1.61 | 5.71 | 9.00 | 12.13 | 10.31 | 38.10 | -0.95 |
| **4x5** | -2.99 | -4.80 | -2.16 | -9.06 | -0.33 | 0.66 | 3.23 | 115.94 |
| **4x6** | -3.98 | -7.21 | 1.43 | 67.45 | 25.50 | 7.26 | 16.67 | 53.96 |
| **4x7** | -4.98 | -9.61 | 3.90 | 74.53 | 27.37 | 12.47 | 31.91 | 135.46 |
| **4x8** | -5.47 | -8.16 | 7.96 | 20.87 | 68.03 | 34.80 | 80.00 | 201.65 |
| **5x6** | -8.33 | -10.00 | 8.57 | 1.38 | 13.60 | 5.70 | 0.00 | 107.68 |
| **5x7** | -9.31 | -10.89 | -7.79 | 13.36 | 11.22 | 0.88 | -3.23 | 94.08 |
| **5x8** | -3.43 | -7.58 | 0.72 | -13.36 | -4.62 | -14.91 | -9.68 | 101.28 |
| **6x7** | -5.24 | -4.76 | -7.79 | 5.88 | -8.59 | 2.18 | -5.56 | -11.18 |
| **6x8** | -8.57 | -6.67 | 1.43 | 12.11 | 14.09 | 2.66 | 11.11 | 11.18 |
| **7x8** | -8.92 | -7.56 | 2.60 | 25.83 | 31.86 | 13.69 | 29.79 | 34.47 |
