# Supplementary material for: Maternal effects, reciprocal differences and combining ability study for yield and its component traits in maize (Zea mays L.) through modified diallel analysis
Source: PeerJ. 2024 Jun 25;12:e17600. doi: 10.7717/peerj.17600 (PMC11212646; doi:10.7717/peerj.17600)
Supplement: Supplemental Information 6 [file peerj-12-17600-s006.docx]

**S6 Table. Better parent heterosis of reciprocal crosses**

| **Crosses** | **DTT** | **DTS** | **NKRPC** | **NKPR** | **CL** | **CG** | **HGW** | **GY** |
| --- | --- | --- | --- | --- | --- | --- | --- | --- |
| **2x1** | -2.60 | -6.00 | 2.99 | 88.50 | 70.89 | 31.74 | 51.06 | 148.04 |
| **3x1** | 4.68 | -5.37 | -7.14 | 28.72 | 33.09 | 13.40 | 64.29 | 3.29 |
| **4x1** | -1.56 | -7.39 | 1.49 | 60.85 | 45.45 | 25.56 | 62.50 | 232.53 |
| **5x1** | -5.73 | -8.38 | -0.72 | 5.68 | 5.68 | 1.97 | -1.61 | 213.40 |
| **6x1** | 3.13 | -2.47 | -8.57 | 6.92 | 13.54 | -3.63 | 1.85 | -12.88 |
| **7x1** | 1.04 | -3.95 | -3.90 | -0.99 | 2.11 | 13.20 | 21.28 | 19.36 |
| **8x1** | -6.77 | -8.87 | 13.11 | 99.47 | 56.60 | 28.93 | 82.50 | 492.12 |
| **3x2** | 4.09 | -1.61 | 0.00 | 9.69 | 7.79 | 9.28 | 19.15 | 51.99 |
| **4x2** | -4.55 | -7.00 | 7.46 | 55.19 | 48.84 | 21.97 | 21.28 | 25.75 |
| **5x2** | -5.05 | -6.00 | 12.95 | 18.59 | 17.82 | 8.33 | 12.90 | 163.49 |
| **6x2** | -2.53 | -4.50 | -1.43 | 24.22 | 9.28 | 5.08 | 11.11 | 18.92 |
| **7x2** | -4.04 | -5.01 | 1.30 | 12.58 | 13.96 | 8.56 | 23.40 | 26.75 |
| **8x2** | -3.54 | -3.51 | 2.99 | 41.00 | 25.46 | 9.58 | 17.02 | 2.32 |
| **4x3** | 8.19 | 0.00 | 0.00 | 12.80 | 7.79 | 14.95 | 40.48 | 21.80 |
| **5x3** | 8.77 | 2.15 | 10.00 | 22.89 | 21.45 | 9.87 | 12.90 | 87.78 |
| **6x3** | 12.28 | 4.84 | 5.71 | 7.27 | 11.89 | 0.97 | -1.85 | 11.39 |
| **7x3** | 10.53 | 3.23 | -6.49 | 17.55 | 20.70 | 5.87 | 19.15 | 71.43 |
| **8x3** | 12.28 | 5.92 | 0.00 | 28.72 | 27.57 | 14.95 | 61.90 | 41.53 |
| **5x4** | -4.98 | -8.65 | 3.60 | 4.15 | 18.81 | 12.50 | 25.81 | 426.42 |
| **6x4** | -4.98 | -8.65 | -2.86 | 6.57 | 13.06 | 2.66 | 12.96 | 20.79 |
| **7x4** | -5.47 | -10.10 | 3.90 | 25.83 | 38.11 | 15.65 | 38.30 | 142.24 |
| **8x4** | -4.98 | -8.16 | 1.49 | 58.49 | 62.04 | 29.91 | 75.00 | 277.07 |
| **6x5** | -6.86 | -9.53 | 10.00 | 1.38 | 7.79 | 0.66 | 1.61 | -24.79 |
| **7x5** | -5.88 | -9.00 | 3.90 | 8.76 | 17.16 | 5.92 | 1.61 | 63.16 |
| **8x5** | -3.92 | -7.58 | -2.16 | -9.68 | 0.00 | -16.89 | -16.13 | 87.02 |
| **7x6** | -7.62 | -8.10 | -5.19 | 22.85 | 20.62 | 6.78 | 0.00 | 35.66 |
| **8x6** | -8.10 | -7.61 | 0.00 | 21.11 | 18.56 | 6.05 | 18.52 | 34.72 |
| **8x7** | -8.45 | -7.56 | 2.60 | 20.20 | 31.23 | 10.27 | 31.91 | 20.30 |
