# Supplementary material for: Maternal effects, reciprocal differences and combining ability study for yield and its component traits in maize (Zea mays L.) through modified diallel analysis
Source: PeerJ. 2024 Jun 25;12:e17600. doi: 10.7717/peerj.17600 (PMC11212646; doi:10.7717/peerj.17600)
Supplement: Supplemental Information 7 [file peerj-12-17600-s007.docx]

**S7 Table : Griffing’s *SCA* effects (Griffing’s *s_ij_*) and partitioned *SCA* of straight (Adj*s_ij_*) and their reciprocal cross (Adj*s_ji_*)**

| **Crosses** | **DTT** | | | **DTS** | | | **NKR** | | | **NKRC** | | |
| --- | --- | --- | --- | --- | --- | --- | --- | --- | --- | --- | --- | --- |
|  | **Griffing’s*s_ij_*^a^** | **Adj*s_ij_*^a^** | **Adj*s_ji_*^a^** | **Griffing’s*s_ij_*** | **Adj*s_ij_*** | **Adj*s_ji_*** | **Griffing’s*s_ij_*** | **Adj*s_ij_*** | **Adj*s_ji_*** | **Griffing’s*s_ij_*** | **Adj*s_ij_*** | **Adj*s_ji_*** |
| 1x2 | **0.24** | 0.08 | 0.41 | **-0.07** | -0.23 | 0.10 | **4.00**^b^** | 2.40** | 5.60** | **0.18** | 0.18 | 0.18 |
| 1x3 | **1.22*** | 2.22** | 0.22 | **-0.36** | 2.14** | -2.86** | **2.61**** | 0.31 | 4.91** | **-0.18** | 0.02 | -0.38* |
| 1x4 | **0.12** | -0.38 | 0.62 | **-0.42** | -0.42 | -0.42 | **0.45** | -2.30** | 3.20** | **0.25** | 0.35* | 0.15 |
| 1x5 | **-1.32*** | -0.65 | -1.98** | **-0.84** | -0.01 | -1.67** | **-0.13** | -2.53** | 2.27** | **-0.19** | -0.49** | 0.11 |
| 1x6 | **-0.23** | -3.57** | 3.10** | **-0.98** | -4.15** | 2.18** | **0.90*** | 3.00** | -1.20** | **0.23** | 1.23** | -0.77** |
| 1x7 | **-0.01** | -1.67** | 1.66** | **-0.38** | -1.88** | 1.12* | **-1.74**** | 0.51 | -3.99** | **0.52**** | 0.72** | 0.32* |
| 1x8 | **-2.88**** | -2.55** | -3.21** | **-2.15**** | -1.48** | -2.82** | **5.82**** | 4.47** | 7.17** | **0.62**** | 0.72** | 0.52** |
| 2x3 | **-0.05** | 1.45* | -1.55** | **-0.63** | -0.63 | -0.63 | **0.72*** | 3.22** | -1.78** | **0.12** | 0.42* | -0.18 |
| 2x4 | **-0.48** | -0.15 | -0.82 | **-0.69** | -0.19 | -1.19* | **1.81**** | 2.81** | 0.81* | **0.36** | 0.56** | 0.16 |
| 2x5 | **-0.92** | -0.76 | -1.09 | **-0.78** | -0.44 | -1.11* | **3.93**** | 2.58** | 5.28** | **0.77**** | 0.32* | 1.22** |
| 2x6 | **-0.51** | -1.01 | -0.01 | **-0.59** | -0.92 | -0.26 | **3.07**** | 3.52** | 2.62** | **-0.16** | 0.24 | -0.56** |
| 2x7 | **-1.11** | -1.11 | -1.11 | **-0.65** | -0.65 | -0.65 | **1.02**** | 3.12** | -1.08** | **0.13** | -0.07 | 0.33* |
| 2x8 | **-0.48** | -0.32 | -0.65 | **-0.59** | -0.92 | -0.26 | **-1.27**** | 1.18** | -3.72** | **-0.38*** | -0.48** | -0.28 |
| 3x4 | **-0.34** | -1.01 | 0.33 | **-0.82** | -1.48** | -0.15 | **-0.29** | -0.89** | 0.31 | **-0.40*** | -0.80** | 0.00 |
| 3x5 | **0.06** | -0.61 | 0.72 | **0.10** | -0.40 | 0.60 | **-0.15** | -6.78** | 6.48** | **0.09** | -0.98** | 1.16** |
| 3x6 | **-0.19** | -2.53** | 2.14** | **-0.21** | -2.55** | 2.12** | **-0.88*** | 0.72* | -2.48** | **0.38*** | 0.08 | 0.68** |
| 3x7 | **-0.13** | -1.30* | 1.04 | **-0.28** | -1.61** | 1.06* | **0.53** | 0.83* | 0.23 | **-0.63**** | -0.63** | -0.63** |
| 3x8 | **0.16** | -1.84** | 2.16** | **0.79** | -0.55 | 2.12** | **2.23**** | -0.62 | 5.08** | **0.56**** | 0.96** | 0.16 |
| 4x5 | **0.12** | 0.79 | -0.55 | **0.37** | 1.70** | -0.96 | **-0.38** | -2.53** | 1.77** | **-0.31** | -0.71** | 0.09 |
| 4x6 | **-0.80** | -0.46 | -1.13* | **-0.61** | -0.11 | -1.11* | **1.06**** | 3.41** | -1.29** | **-0.28** | 0.02 | -0.58** |
| 4x7 | **-1.40*** | -1.23* | -1.57** | **-2.01**** | -1.84** | -2.17** | **3.62**** | 3.12** | 4.12** | **0.90**** | 0.90** | 0.90** |
| 4x8 | **-1.28*** | -1.44* | -1.11 | **-1.44*** | -1.44** | -1.44** | **3.54**** | 4.20** | 2.87** | **0.13** | 0.57** | -0.30 |
| 5x6 | **-1.90**** | -2.40** | -1.40** | **-1.86**** | -2.03** | -1.69** | **-0.32** | -0.32 | -0.32 | **0.87**** | 0.77** | 0.97** |
| 5x7 | **-2.01**** | -3.17** | -0.84 | **-1.76**** | -2.42** | -1.09* | **1.03**** | 1.78** | 0.28 | **-0.24** | -1.14** | 0.66** |
| 5x8 | **0.79** | 0.95 | 0.62 | **-0.69** | -0.69 | -0.69 | **-3.16**** | -3.76** | -2.56** | **-0.34** | -0.14 | -0.54** |
| 6x7 | **0.08** | 0.91 | -0.76 | **0.27** | 1.43** | -0.90 | **-1.23**** | -4.48** | 2.02** | **-0.82**** | -1.02** | -0.62** |
| 6x8 | **-1.13** | -1.30* | -0.96 | **-0.84** | -0.51 | -1.17* | **1.78**** | 0.48 | 3.08** | **0.08** | 0.18 | -0.02 |
| 7x8 | **-0.57** | -0.73 | -0.40 | **-0.57** | -0.57 | -0.57 | **3.43**** | 4.28** | 2.58** | **0.87**** | 0.87** | 0.87** |
| **S.Ed*s_ij_*** | **0.16** | | | **0.53** | | | **0.33** | | | **0.16** | | |

Cont.,

| **Crosses** | **CL** | | | **CG** | | | **HGW** | | | **GY** | | |
| --- | --- | --- | --- | --- | --- | --- | --- | --- | --- | --- | --- | --- |
|  | **Griffing’s*s_ij_*** | **Adj*s_ij_*** | **Adj*s_ji_*** | **Griffing’s*s_ij_*** | **Adj*s_ij_*** | **Adj*s_ji_*** | **Griffing’s*s_ij_*** | **Adj*s_ij_*** | **Adj*s_ji_*** | **Griffing’s*s_ij_*** | **Adj*s_ij_*** | **Adj*s_ji_*** |
| 1x2 | **2.40**** | 0.51 | 4.29** | **0.19**** | -0.05 | 0.44** | **2.83**** | -1.67** | 7.33** | **22.65**** | 9.49** | 35.81** |
| 1x3 | **1.19**** | -0.10 | 2.48** | **0.06** | -0.04 | 0.16** | **4.64**** | 2.39** | 6.89** | **-8.98**** | -11.31** | -6.66** |
| 1x4 | **0.37** | -0.35 | 1.09** | **0.08** | 0.00 | 0.17** | **2.08**** | 1.08** | 3.08** | **0.87** | 8.15** | -6.42** |
| 1x5 | **-1.22**** | -2.43** | -0.02 | **-0.09*** | -0.33** | 0.15** | **-1.33**** | -3.08** | 0.42 | **-4.26** | -10.73** | 2.22 |
| 1x6 | **0.50** | 0.77 | 0.24 | **-0.11*** | 0.05 | -0.26** | **-0.48** | -0.98* | 0.02 | **-2.80** | 6.35* | -11.96** |
| 1x7 | **-0.91*** | 0.39 | -2.21** | **0.10*** | -0.05 | 0.25** | **0.64** | 0.14 | 1.14** | **3.93** | 17.50** | -9.64** |
| 1x8 | **2.00**** | 1.63** | 2.38** | **0.41**** | 0.29** | 0.53** | **5.64**** | 3.39** | 7.89** | **30.52**** | 15.96** | 45.07** |
| 2x3 | **0.07** | 0.81* | -0.68* | **0.10*** | 0.24** | -0.05 | **0.48** | 1.73** | -0.77 | **3.38** | -6.87** | 13.64 |
| 2x4 | **0.72** | 1.15** | 0.28 | **0.08** | 0.18** | -0.02 | **-0.33** | 1.42** | -2.08** | **-2.94** | 2.89 | -8.77** |
| 2x5 | **1.34**** | 0.58 | 2.11** | **0.27**** | 0.15** | 0.39** | **2.02**** | 0.27 | 3.77** | **16.34**** | -5.56 | 38.25** |
| 2x6 | **0.50** | 1.10** | -0.10 | **0.07** | 0.09* | 0.05 | **1.11*** | 0.86* | 1.36** | **-0.47** | -1.51 | 0.57 |
| 2x7 | **0.69** | 1.62** | -0.24 | **0.04** | 0.07 | 0.01 | **0.98*** | 1.48** | 0.48 | **-15.24**** | -22.90** | -7.59** |
| 2x8 | **-0.97*** | 0.26 | -2.19** | **0.00** | 0.21** | -0.22** | **-1.27**** | -0.27 | -2.27** | **-9.32**** | -10.51** | -8.13** |
| 3x4 | **-0.89*** | -0.90** | -0.89* | **0.03** | -0.07 | 0.12** | **-0.52** | -0.52 | -0.52 | **-2.34** | -2.34 | -2.34 |
| 3x5 | **0.19** | -2.44** | 2.83** | **0.13**** | -0.20** | 0.47** | **2.08**** | -0.17 | 4.33** | **10.81**** | -5.34 | 26.96** |
| 3x6 | **0.13** | -0.18 | 0.45 | **-0.08** | -0.05 | -0.11** | **-1.83**** | -2.08** | -1.58** | **-1.01** | 2.66 | -4.68 |
| 3x7 | **0.14** | -0.61 | 0.89* | **-0.13**** | -0.17** | -0.09* | **0.05** | 0.05 | 0.05 | **5.27*** | -5.25* | 15.79** |
| 3x8 | **0.73*** | -0.32 | 1.78** | **0.28**** | 0.19** | 0.37** | **2.30**** | -0.20 | 4.80** | **5.49*** | -3.65 | 14.63** |
| 4x5 | **0.59** | -0.86* | 2.04** | **0.26**** | -0.01 | 0.53** | **3.02**** | -0.48 | 6.52** | **8.07**** | -17.45** | 33.60** |
| 4x6 | **1.14**** | 2.05** | 0.24 | **-0.01** | 0.09* | -0.10* | **1.11*** | 1.61** | 0.61 | **5.24*** | 12.12** | -1.64 |
| 4x7 | **2.22**** | 1.46** | 2.99** | **0.19**** | 0.12** | 0.26** | **1.98**** | 1.23** | 2.73** | **30.56**** | 29.32** | 31.81** |
| 4x8 | **1.87**** | 2.19** | 1.55** | **0.36**** | 0.44** | 0.27** | **4.48**** | 4.98** | 3.98** | **2.71** | -2.64 | 8.05** |
| 5x6 | **0.53** | 0.97** | 0.09 | **0.16**** | 0.28** | 0.05 | **0.70** | 0.45 | 0.95* | **7.83**** | 35.30** | -19.63** |
| 5x7 | **0.58** | 0.13 | 1.03** | **0.03** | -0.08 | 0.15** | **0.33** | -0.42 | 1.08** | **9.37**** | 15.04** | 3.69 |
| 5x8 | **-1.18**** | -1.53** | -0.83* | **-0.52**** | -0.48** | -0.57** | **-4.67**** | -3.67** | -5.67** | **-12.55**** | -11.38** | -13.72** |
| 6x7 | **-1.55**** | -3.67** | 0.58 | **-0.11*** | -0.20** | -0.01 | **-1.58**** | -2.33** | -0.83* | **-8.41**** | -18.12** | 1.30 |
| 6x8 | **0.69** | 0.36 | 1.01** | **0.21**** | 0.14** | 0.28** | **1.92**** | 0.92* | 2.92** | **7.74**** | 2.86 | 12.62** |
| 7x8 | **2.03**** | 2.08** | 1.99** | **0.34**** | 0.41** | 0.27** | **1.80**** | 1.55** | 2.05** | **-1.86** | 0.74 | -4.46 |
| **S.Ed*s_ij_*** | **0.34** | | | **0.04** | | | **0.39** | | | **2.49** | | |

^a^Griffing’s*s_ij_* – The *SCA* effect estimated following Griffing’s procedure, Adj*s_ij_* – The adjusted *SCA* effect of straight cross after partitioning, Adj*s_ji_* – the adjusted *SCA* effects of reciprocals after partitioning.

^b^* and ** indicate significance at 0.05 and 0.01 probabilities, respectively.
